# COVID-19 in European Soccer: A Public 2-Year Comparison of COVID-19 Case Management and Case Characteristics between the 1st Bundesliga, La Liga, Serie A and the Premier League

**DOI:** 10.3390/life12081220

**Published:** 2022-08-11

**Authors:** Jan-Niklas Droste, Robert Percy Marshall, Stephan Borte, Sebastian Seyler, Helge Riepenhof

**Affiliations:** 1 Medical Department, RasenBallsport Leipzig GmbH, 04177 Leipzig, Germany; 2 Center for Rehabilitation and Sports Medicine, BG Klinikum Hamburg, 21033 Hamburg, Germany; 3 Department of Laboratory Medicine, Hospital St. Georg Leipzig, 04129 Leipzig, Germany; 4 Red Bull Athlete Performance Center Thalgau, 5303 Salzburg, Austria

**Keywords:** professional soccer, pandemic, prevention and control, virus transmission, disease burden

## Abstract

To evaluate the extent and characteristics of COVID-19 cases in relation to environmental COVID-19 incidences in the four best European soccer leagues (Bundesliga, Premier League, Serie A and La Liga) from the first of January 2020 until the end of January 2022. **Methods**: A retrospective evaluation of all publicly available COVID-19 cases in the studied cohorts was performed. The 14-day case incidences from epidemiological national data were used as reference values. The leagues studied are the Bundesliga (Germany), Premier League (Great Britain), Serie A (Italy) and La Liga (Spain). For all cases, the duration of time loss and date of case notification were recorded. **Results**: League-specific mean time loss due to disease or quarantine per COVID-19 case differs significantly between La Liga (11.45; ±5.21 days) and the other leagues studied (Bundesliga 20.41; ±33.87; *p* 0.0242; Premier League 17.12; ±10.39; *p* 0.0001; Serie A 17.61; ±12.71; *p* < 0.0001). A positive correlation between 14-day national incidence with COVID-19 disease occurrence in soccer leagues was found for all leagues studied. The correlations were strong in the Bundesliga (r 0.5911; CI 0.4249–0.7187; *p* < 0.0001), Serie A (r 0.5979; CI 0.4336–0.7238; *p* < 0.0001) and La Liga (r 0.5251; CI 0.3432–0.6690; *p* < 0.0001). A moderate correlation was found for the Premier League (r 0.3308; CI 0.1147–0.5169; *p* 0.0026). Odds ratios for altered environmental case risk in the cohorts studied could be calculated for four different national COVID-19 incidence levels (<50/100.000 to >500/100.000). A trend towards shorter COVID-19 case duration in the second half of 2021 was shown for all leagues studied. **Conclusions**: There was a significantly lower mean time-loss caused by a COVID-19 infection for cases occurred in La Liga compared with the other three leagues studied. For all four leagues studied, a positive, significant correlation of national environmental COVID-19 incidence level and the incidence of COVID-19 cases in the cohort of a football league was found.

## 1. Introduction

The severe acute respiratory syndrome coronavirus 2 (SARS-CoV-2) resulted in huge impacts on world society including professional sports as well as everyday activities at the beginning of 2020 [1]. This virus had as of April 2022 caused more than 500 million confirmed infections and has been associated with more than 6 million deaths in the past two years, being responsible for the coronavirus disease 2019 (COVID-19) [2,3,4].

SARS-CoV-2 is a virus that can cause a COVID-19 infection, spread by aerosol droplets, that primarily affects the upper respiratory tract with pulmonal complications [5]. Additional symptoms are fever and chills as well as myalgic, neurological, renal and gastro-intestinal complaints [6,7]. Severe complications involve the heart with myocarditis and arrythmias [8]. Typical recovery times last about 14 to 21 days [9,10].

Long COVID-19 is a pathological state with symptoms like dizziness, breathlessness and fatigue that last longer than four weeks after first symptom onset [11,12]. Post-infectious (long-term) sequelae have been frequently reported after acute viral diseases [13,14,15,16,17,18]. Similar to this, patients who recover from COVID-19 often complain about long-lasting pulmonary, cardiovascular, neurological, hematological and neuro-psychiatric complications [19,20]. In the general population, 20–30% of COVID-19 patients exhibit symptoms for 5 weeks, and 10% complain of disabilities lasting for more than 12 weeks [21,22]. Information is still lacking about the incidence and prevalence of long COVID-19 in athletes [23].

Being reported in China in November 2019, the virus spread quickly across the globe, reaching Europe in February 2020 [24]. This pandemic situation stopped nearly every public event including all sporting events [25,26]. The German Bundesliga introduced hygiene principles that would allow for restarting training and competition, with other leagues following [26,27,28,29,30]. Following this example, sports all over the world were allowed to restart their schedules under strict conditions and initially under exclusion of the audience [30,31,32,33].

Early European epidemiologic studies demonstrated that training and competition continued with nearly no infections across teams even when the viral burden of the surrounding population rose [34]. Strict hygiene plans and repetitive testing (PCR or lateral-flow antigen) were able to keep in-team infection rates at low levels [35,36]. Sports were performed in a so-called bubbles with no outside contact [37,38]. Probably the most severe measure was enacted in the United States, where the National Basketball Association finished its season with its teams living and competing in an enclosed leisure park for several weeks [39,40,41]. Despite common goals for a safe return to sports practice among different sports leagues around the world, there was high variation in the hygienic measures and non-pharmaceutical interventions that were implemented [42,43].

The risk of virus transmission from COVID-19-infected athletes during a soccer match has been reported to very low due to very short contact times [44]. No large-scale outbreaks within these settings have been reported. However, laboratory screenings in the German league detected an antibody prevalence that was eight to ten times higher than documented positive PCR tests. These results suggest that there is a much higher infection rate than reported, possibly due to incorrect testing or mild and undetected infections [45].

Interestingly, in countries with higher infection rates within the general population, significantly higher infection rates among professional athletes were reported [30].

Primarily, due to cardiac complications in SARS-CoV-2-infected athletes, various return-to-sport algorithms were introduced with an initial minimal proposed dropout time of 14 days in asymptomatic athletes and longer in athletes with infectious symptoms [46,47,48,49,50]. With vaccination against SARS-CoV-2 being developed and introduced into the public, the infectious disease burden was reduced in the general population as well as in athletes in the first half of 2021 [51,52,53]. Complication rates dropped, with a majority of mild and asymptomatic courses of COVID-19 being reported. This changed proposed return-to-sport algorithms for vaccinated individuals to recommended dropout times of 5 days in athletes with mild symptoms and 3 days in asymptomatic athletes [54,55,56,57].

The disease burden of COVID-19 on professional sports remains unclear. Therefore, this research aims to elucidate the direct impacts of COVID-19-inflicted absences in Europe’s four biggest football leagues (German Bundesliga, English Premier League, Spanish La Liga, Italian Serie A) between 01.01.2020 and 31.01.2022. Publicly accessible data were evaluated and correlated with each team’s national infection rates, incidence and time courses. This research is to help professional sports with future handling of infectious outbreaks comparable with COVID-19.

Our work shares new insights into demographical differences in infection rates of the different leagues as well changes in infection rates over time’s course. Due to earlier research that was conducted in single countries and athlete populations only, we anticipate that our research will provide a possible connection between infection rates within the leagues and infection rates in the general local population.

## 2. Materials and Methods

### 2.1. Population and Data Collection

The 78 first division soccer teams of Germany (Bundesliga), Great Britain (Premier League), Italy (Serie A) and Spain (La Liga) were investigated for this retrospective, observational cohort study using publicly available databases, websites and press releases [58,59]. Data collection was conducted on 1 March 2022. The data collection was performed by two authors and supervised as well as cross-checked be the senior author. For all players on the above-mentioned teams in the 2019/2020, 2020/2021 and 2021/2022 seasons, retrospective lost-time data were analyzed, and those lost-time events were included that were causally related to the COVID-19 pandemic (quarantine measures as well as COVID-19 infections). Teams that did not publish documented COVID-19 infections over the three-season period were excluded from the analysis. All COVID-19 associated absences between 1 January 2020 and 31 January 2022 were included in the analysis. In addition, national epidemiologic data (weekly infection counts, 14-day notification rate of newly reported COVID-19 cases per 100,000 population by day, updated per week nationwide) were included in the analysis as a reference group using publicly available data from the European Centre for Disease Prevention and Control (ECDC) [60]. For the Germany, Italy and Spain data pool, the ECDC data are based on the Tessy COVID-19 program. For the UK data pool, data are based on national epidemiological surveys. All data used herein are freely available to the public and were processed anonymously, thereby not requiring ethical permissions. The study was conducted in accordance with the Helsinki declaration. There was no financial support or conflict of interest.

### 2.2. Definition of Cases, Quarantine and Severity

As part of the data collected on COVID-19-associated time lost in the soccer cohorts studied, time lost was differentially broken down as isolation measure/quarantine or illness/case. Measure-associated downtime was defined and determined as absence from regular training and game play. In the case of illness/cases, case severity was introduced using ordinal grouping. Since isolation measures were ordered by many European countries for 14 days at the beginning of the pandemic, a downtime of maximum 14 days is classified as a mild illness in the context of this study. The authors acknowledge that isolation time as well as other non-pharmaceutical measures did vary widely among countries and over time. Downtime of more than 14 to a maximum of 28 days was classified as moderate. Cases with a downtime of more than 28 days were classified as severe/protracted cases. Those three categories were adopted from the International Olympic Committee consensus statement on injury and illness classification [61].

### 2.3. Statistical Analysis

Descriptive analysis of COVID-19-related case and quarantine data was made by the grouped calculation of percents and means including SD and 95% CIs. Normal distribution was tested for metric data with the Shapiro–Wilk test. Data were then considered non-normally distributed; therefore, the Kruskal–Wallis test was performed to compare medians. Dunn’s multiple-comparisons tests was performed. For all statistics, *p* values < 0.05 were regarded as significant.

Correlations between cohort data and epidemiological data were analyzed by calculating Spearman’s rank correlation coefficient for the nonparametric correlations. Two-tailed *p* values < 0.05 were regarded as significant. The thresholds for the correlation coefficients were 0.1 (small), 0.3 (moderate), 0.5 (large), 0.7 (very large) and 0.9 (extremely large) [62,63].

Odds ratios were calculated using 95% CIs and *p* values < 0.05 to observe the relationships between epidemiological 14-day case notification rate and cohort case risk.

Case severity fraction of total analysis was performed using 95% CIs. Additional chi-square goodness-of-fit tests were performed to compare observed distribution with theoretical distribution. The overall studied period of time was set as expected frequency (outcome) and compared with sub-period expected outcomes (1 January 2020–31 December 2020; 1 January 2021–last day of week 26 2021; first day of week 27 2021 until end of observation period). For all calculations, *p* values < 0.05 were regarded as significant.

Statistical analysis was conducted using Graphpad Prism 9 (GraphPad Software; 2365 Northside Dr.; Suite 560; San Diego, CA, USA).

## 3. Results

### 3.1. Descriptive Statistics Downtime

**Figure 1:**
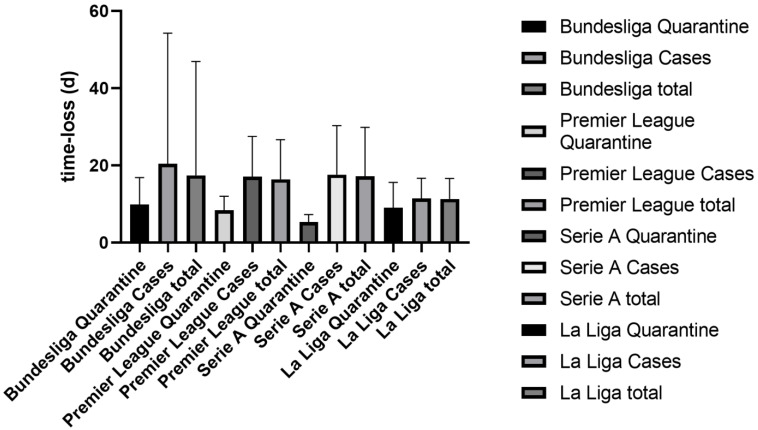
Mean (+95% CI) time losses in days (d) per COVID-19 case, quarantine measure and total of both differentiated among the four leagues studied.

During the period studied, we collected 677 COVID-19-associated lost-time events with 10.815 lost-time days in the four leagues. An additional 682 downtime days were due to quarantine measures, and 10.133 were due to COVID-19. The variation among leagues is shown in Table 1. The average length of COVID-19-associated quarantine did not differ significantly between Bundesliga (mean 9.865; ±6.985), Premier League (mean 8.375; ±3.662), Serie A (mean 5.333; ±1.936) and La Liga (mean 9.0; ±6.603). The average time lost in days due to COVID-19 infection showed no significant differences between Bundesliga (mean 20.41; ±33.87), Premier League (mean 17.12; ±10.39) and Serie A (mean 17.61; ±12.71). However, the mean COVID-19 lost time in La Liga (mean 11.45; ±5.206) was significantly different from the means for all other leagues (Bundesliga *p* 0.0242; Premier League *p* 0.0001; Serie A *p* < 0.0001). League-specific differences are shown in Figure 1.

### 3.2. Correlation 14-Day National Incidence with Disease Occurance in Soccer Leagues

The 14-day incidence per 100,000 population in Germany correlates strongly (r = 0.5911; 95% CI 0.4249–0.7187; *p* < 0.0001) with the case incidence in the Bundesliga in the associated calendar week. In the Premier League, there is a moderate correlation of case probability with 14-day incidence per 100,000 population in the United Kingdom (r = 0.3308; 95% CI 0.1147–0.5169; *p* 0.0026). There are also strong correlations with 14-day incidence per 100,000 population in Serie A (r = 0.5979; 95% CI 0.4336–0.7238; *p* < 0.0001) and La Liga (r = 0.5251; 95% CI 0.3432–0.6690; *p* < 0.0001) in the respective countries.

### 3.3. Odds Ratios Surrounding Incidence with Probability of Occurrence of COVID-19 Cases in the Leagues

In the Bundesliga, the probability of a COVID-19 case in the cohort increases by a factor of 6.65 when the ambient incidence increases from <50 to 50 to 199 (95% CI 1.7248–25.6390; z 2.752; *p* 0.0059). At higher incidences, the case probability further increases between ambient incidence levels (<50; 50–199; 200–499; ≥500) without being significant. The probability of the occurrence of COVID-19 disease when ambient incidence increases from <50 to 200–499 increases by 8.4444 in the Bundesliga (95% CI 2.1834–32.6587; z = 3.092; *p* < 0.002) and a 47.5 increase when increasing to ambient incidences from <50 to ≥500 (95% CI 4.6620–483.9645; z = 3.26; *p* 0.0011).

In the Premier League, the probability of COVID-19 case occurrence in the cohort does not increase significantly between incidence levels (<50; 50–199; 200–499; ≥500). Increasing the ambient incidence from below 50 to 200–499 significantly increases the risk of COVID-19 cases by a factor of 28 (95% CI 2.8204–277.9723; z = 2.845; *p* < 0.004). Compared with ambient incidence < 50, in the Premier League, ambient incidence > 500 increases case risk by a factor of 13.2632 (95% CI 1.5781–111.4692; z = 2.38; *p* < 0.0173).

In Serie A, between incidence levels (<50; 50–199; 200–499; ≥500), with the exception of an increase in ambient incidences from 50–199 to 200–499 by a factor of 6.16 (95% CI 1.7277–21.8364; z = 2.816; *p* < 0.0049), within-cohort increases in case probability are nonsignificant. An increase in ambient incidence from <50 to 200–499 significantly increases the risk by 9.5333 (95% CI 2.4209–37.5418; z = 3.224; *p* < 0.0013). An ambient incidence increase from <50 to ≥500 leads to an increased risk of Serie A cases by a factor of 56.0769 (95% CI 2.8661–1097.1816; z = 2.654; *p* < 0.008).

**Figure 2:**
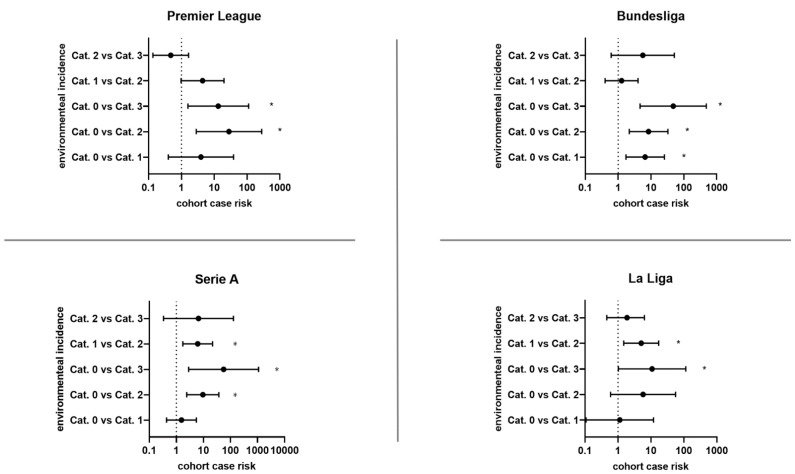
Odds ratios (ORs) for different environmental 14-day incidence levels in the league-specific country and case risk in the related observed cohort (league). Environmental incidence levels are classified into four categories (Cat.): Cat. 0 = incidence < 50 per 100,000 residents; Cat. 1 = incidence of 49–199 per 100,000 residents; Cat. 2 = incidence of 200–499 per 100,000 residents; Cat. 3 = incidence > 500 per 100,000 residents. 95% confidence intervals (CI) are shown with lower limit and upper limit. *p*-value estimation is set with a significance < 0.05. Significant odds ratios are highlighted (*).

Similarly, in La Liga, there is no significant increase in case probability within the cohort between incidence levels (<50; 50–199; 200–499; >/=500) except for an increase in ambient incidence from 50–199 to 200–499 by a factor of 5.0769 (95% CI 1.4951–17.2399; z = 2.605; *p* < 0.0092). Increased ambient incidence to 200–499 compared with <50 does not significantly increase case probability. Compared with ambient incidence < 50, ambient incidence > 500 significantly increases case probability by a factor of 10.8333 (95% CI 1.0281–114.1550; z = 1.983; *p* < 0.0474). League-specific differences are shown in Table 2 and Figure 2. Total values for all four leagues are shown in Adnex S1.

### 3.4. Severity of Downtime in COVID-19 Infection

In the Bundesliga, 59% (95% CI 51.248–67.139) of cases resulted in downtime of less than 14 days (mild). Downtime of 14–28 days occurred in 27% (95% CI 20.016–34.357) of cases (moderate). Downtime > 28 days occurred in 14% (95% CI 9.24–20.617) of cases (long). In the 2020 observation period, case severity differed significantly from the overall observation period, with only 48% mild cases, 20% moderate cases, and 32% long cases (chi-square 8.644; DF 2; *p* (two-tailed) 0.0133). In the first half of 2021, as well as for the period from the second half of 2021 on, there was no significant deviation in the case severity distribution compared with the overall observation period.

In the Premier League, 49% (95% CI 38.82–59.048) of cases resulted in a downtime of less than 14 days (mild). Downtime of 14–28 days occurred in 43% (95% CI 33.577–53.636) of cases (moderate). Downtime > 28 days occurred in 8% (95% CI 3.818–15.194) of cases (long). The case severity distribution did not differ significantly from the overall observation period for the observation period 2020, the first half of 2021, and from the second half 2021 onward.

In Serie A, 51% (CI 45.169–57.690) of cases resulted in downtime of less than 14 days (mild). Downtime of 14–28 days occurred in 39% (CI 32.668–44.868) of cases (moderate). Downtime > 28 days occurred in 10% (CI 6.784–14.39) of cases (long). In the 2020 observation period, case severity differed significantly from the overall observation period, with only 28.21% mild cases, 57.69% moderate cases and 14.10% long cases (chi-square 16.25; DF 2; *p* (two-tailed) 0.0003). This significant difference also occurred in the first half of 2021, with 35.29% mild cases, 47.06% moderate cases and 17.65% long cases (chi-square 6.298; DF 2; *p* (two-tailed) 0.0429) as well as for the period from the second half of 2021 with 75% light cases, 21.43% moderate cases and 3.571% long cases (chi-square 26.14; DF 2; *p* (two-tailed) 0.0001). That is, there was a significant deviation in the case severity distribution compared to the overall observation period.

**Figure 3:**
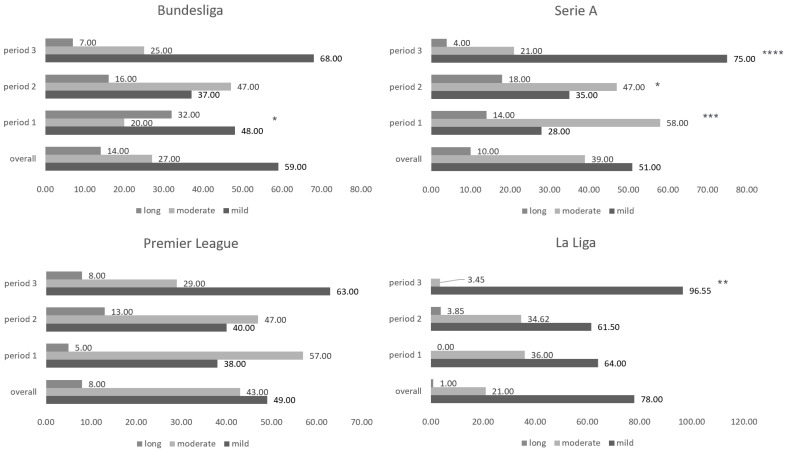
Relative case severity/duration for the overall period studied in this research. Case severity/duration is categorized as mild (<14 days of time loss), moderate (14–28 days of time loss), or long (>28 days of time loss). Significant differences between single periods and the overall studied are highlighted by * (* = *p* ≤ 0.05, ** = *p* ≤ 0.01, *** = *p* ≤ 0.001, **** = *p* ≤ 0.0001).

In La Liga, 78% (CI 70.618–84.834) of cases resulted in downtime of less than 14 days (mild). Downtime of 14–28 days occurred in 21% (CI 14.489–28.518) of cases (moderate). Downtime > 28 days occurred in 1% (CI 0.0041–4.359) of cases (long). The case severity distribution did not differ significantly from the overall observation period for the observation period 2020 and the first half of 2021. In the observation period from the second half of 2021 onward, case severity significantly deviated from the overall observation period, with 96.55% mild cases, 3.448% moderate cases and 0% long cases (chi-square 11.65; DF 2; *p* (two-tailed) 0.003). Relative case severity and duration for the overall period and subanalysis studied in this research are shown in Figure 3 and Table 3.

## 4. Discussion

This research aims to elucidate direct impacts of COVID-19 on absences and time losses in Europe’s four biggest football leagues. Significant differences in the number of documented cases were apparent across the leagues studied for the inspected time interval. Because of the methodology using publicly available data on COVID-19 cases and quarantine measures, there are justified concerns about the possible risk of bias. Due to missing data or over- and under-interpretation, as well as the national differences regarding quarantine and isolation times as well as non-pharmaceutical measures, we did not perform a statistical test or comparison of the case occurrence probability among the leagues [64]. SARS-CoV-2 and COVID-19-related measures did not vary only between nations and leagues; there was also a responsive change over time.

Based on the methodology used in this study, a high number of documented cases was collected, allowing a comparison of case-specific metrics among leagues despite limitations in methodology. Average quarantine time, unlike disease-related downtime, did not differ significantly among leagues. In the context of the COVID-19 pandemic, the risks of sports exposure during and after infection with COVID-19 were extensively highlighted, and there were scientific studies on the topic early on. Cardiac complications in particular pose an increased risk for athletes [65]. In addition, pulmonary and multi-organ complications have been reported [66]. It needs to be highlighted that downtime does not necessarily mean infection. The term “downtime” refers to the athlete being not available for team training and/or competition. This could be due to infection severity for the maximum number of days as well as official regulations for the minimum number of days [67,68,69,70].

Testing regimes in different countries vary significantly [71]: Some clubs bought PCR machines to test themselves, whereas others have cooperation with laboratories. PCR tests as well as different sensitive lateral flow tests have been introduced [72]. Leagues and clubs were allowed to choose between them at different times. Officially documented infection rates are therefore potentially related to the testing protocols as well as infection severity.

In the study conducted here, the significant difference between the average return to sport times between La Liga (Spain) and the three other leagues investigated was particularly striking. Athletes playing in La Liga who were infected with SARS-CoV-2 missed fewer training days than players in any other league that was compared. A possible reason for this could have been that the vast number of infections occurred during the second or third time periods with rising vaccination status and reduced recovery times. This hypothesis was, however, not consistent with our data showing increasing infection rates in all leagues over time with no differences (Table 3). Another explanation for the reduced dropout times in Spain could have been more relaxed return-to-sports algorithms. In the context of sports medicine practice, several recommendations were proposed for the safe return of athletes after COVID-19 infection. Professional athletes are not typically identified as high-risk patients. They are young, healthy and, due to league regulations, frequently checked by medical professionals [73]. Therefore, there are reasonable factors that allow professional athletes to return to sports in a more progressive manner than other amateur athletes or general populations [74,75,76]. However, this aspect can be discussed critically and controversially. In this regard, screening examinations as well as recommended sports abstinence periods were elaborated. A disease-related sports break of at least two weeks is recommended [46,77]. Therefore, the average downtime per COVID-19 infection of only 11.45 days in La Liga (±5206) needs to be highlighted as differing significantly from the other leagues’ dropout times after infections. Due to the methodology used, no causal reasons for these deviations can be investigated.

It has already been demonstrated that COVID-19-associated psychosocial characteristics varied among European countries. In particular, countries that had high rates of infection and deaths at the onset of the pandemic, including Spain and Italy, exhibit a more complex response pattern including anxiety and reluctance [78]. Multifactorial effects, which may also be influenced by cultural characteristics, have already been demonstrated among different soccer leagues for injuries [79]. Therefore, it might be reasonable that countries with less strict restrictions regarding the virus transmission are facing COVID-19 infections with less fear and are more liberal in the process of returning to sports despite possible long-term complications. Further research is needed to clarify causal relationships.

A correlation between case occurrence probability and population incidence was demonstrated for all four leagues studied. The Premier League documented the lowest infection rates, followed by La Liga and the Bundesliga, and the most cases were recorded in Serie A. Possible explanations for the differences in the magnitude of the correlation may be individual national containment measures but also variations in COVID-19 prevention approaches across the leagues. In this context, it needs to be explained that relative to hygienic response levels within Europe, the UK has been rated most progressive, followed by Germany, Italy and eventually Spain with the least strict responses to the pandemic [80]. Another possible reason for lower infection rates could be different testing protocols. This could eventually lead to athletes continuing to train and compete in matches while being infected but not tested because they are not symptomatic.

As part of the statistical analysis, it was also possible to examine a risk of grouped environmental incidence with a case occurrence probability in each league. As expected, the risk of COVID-19 cases within soccer leagues increases with increasing ambient incidence. Leagues attempted to prevent the penetration of cases into the cohorts studied, especially at the beginning of the pandemic, using bubbles [81]. For the future, the finding is that there is a direct dependency of the risk of infection within the cohort despite general containment measures as well as sport- and club-specific prevention concepts. In other words, there seems to be a direct correlation and influence of the surrounding infection rates despite the specific hygienic rules implemented in a given league setting. In the authors’ own clinical experience, most infection cases were caused during holidays or meeting friends and family during off time. Therefore, it is reasonable that only extreme measurements like nationwide lockdowns or specific club-related measurements like locking athletes in a leisure park or their training grounds really led to a significantly low incidence in infections. As soon as contact with the outer world was allowed, infection rates were related to the local epidemiological infection rates. National team travels were only a minor factor initially when COVID hygiene rules differed internationally.

The Bundesliga, Serie A and La Liga showed significant differences in case severity distribution during different time periods of the pandemic. All leagues show a significant increase in mild cases in period 3. This fact could be due to increased vaccination as well as recovery rates with the pandemic slowly becoming endemic [82,83]. Interestingly, the Premier League has been the major soccer league known for the lowest vaccination rates compared with the Spanish, German and Italian leagues [84]. Since methodologically no individual disease data are available, case severity can only be derived using downtime. This is a methodological limitation of the conducted study. Possible factors for varying case severity in follow-up could be mutations of the SARS-CoV-2 virus, increasing vaccination rates in the studied cohort over the course of the year 2021 or increased medical competence in the assessment of COVID-19 infection risk and disease severity [85,86]. Again, causal relationships cannot be shown on an individual basis due to the methodology. Interestingly, the temporal severity changes in the Premier League cohort do not show significant divergence from the overall observation period. We do not have an explanation for this phenomenon. In general, however, it can be assumed that changes in downtime and case severity are multifactorial.

Taken together, our data indicate there is a direct correlation between national incidence rates and COVID-19-associated absence times in professional European football leagues. It appears that only massive restrictions similar to the NBA leisure park bubble in 2020 that prohibited any contact between athletes and their surroundings led to a significant reduction in infection rates. As soon as athletes were allowed private contacts during their non-training times, infection rates correlated with the general population. Therefore, the following hypothesis can be stated: eventually, an infectious situation in the local population will find its way into a so-called bubble. This cannot be avoided unless any contact with the outer world is prohibited. Focus should, therefore, remain on identifying infectious athletes early enough and maintaining hygiene rules within athletes’ settings to avoid spreading infection in the bubble.

## 5. Conclusions

Significant differences in COVID-19-associated case-specific downtime among the four leagues studied were shown. Furthermore, a positive correlation between the ambient incidence of the respective country and the case occurrence probability in the associated league was shown. Additionally, the Bundesliga, La Liga, and Serie A show changes in downtime as the pandemic progresses towards shorter case duration. This fact was not demonstrated in the Premier League. Multiple factors could be linked to this finding such as regulatory measures and vaccination rates as well as multiple viral variants mutating during the studied period. Based on the methodology, the above points could be identified. However, there are methodological limitations that did not allow for identifying causality with individual factors. Further studies are required to substantiate the proven differences causally.

## Figures and Tables

**Table 1:** Descriptive statistics of all analyzed COVID-19-related time losses. Columns contain case-specific, quarantine-specific and total (cases and quarantine measures) per league studied.

	Bundesliga			Premier League			Serie A			La Liga		
	Quarantine	Cases	Total	Quarantine	Cases	Total	Quarantine	Cases	Total	Quarantine	Cases	Total
**Number of values**	52	143	195	8	90	98	9	241	250	6	128	134
**Minimum**	2	3	2	3	6	3	3	3	3	3	3	3
**Maximum**	42	315	315	15	67	67	8	104	104	22	45	45
**Range**	40	312	313	12	61	64	5	101	101	19	42	42
**Mean**	9.8650	20.4100	17.4100	8.3750	17.1200	16.4100	5.3330	17.6100	17.1700	9.0000	11.4500	11.3400
**Std. Deviation**	6.9850	33.8700	29.5300	3.6620	10.3900	10.2900	1.9360	12.7100	12.6900	6.6030	5.2060	5.2700
**Std. Error** **(of Mean)**	0.9686	2.8330	2.1150	1.2950	1.0950	1.0390	0.6455	0.8186	0.8026	2.6960	0.4601	0.4553
**Lower 95% CI** **(of mean)**	7.9210	14.8100	13.2400	5.3130	14.9500	14.3500	3.8450	16.0000	15.5900	2.0710	10.5400	10.4400
**Upper 95% CI** **(of mean)**	11.8100	26.0100	21.5800	11.4400	19.3000	18.4700	6.8220	19.2200	18.7500	15.9300	12.3600	12.2400
**Sum (days)**	513	2919	3395	67	1541	1608	48	4244	4292	54	1466	1520

**Table 2:** Odds ratios (ORs) for different environmental 14-day incidence levels in the league-specific country and case risk in the related observed cohort (league). Environmental incidence levels are classified into four categories (Cat.): Cat. 0 = incidence < 50 per 100,000 residents; Cat. 1 = incidence of 49–199 per 100,000 residents; Cat. 2 = incidence of 200–499 per 100,000 residents; Cat. 3 = incidence > 500 per 100,000 residents. 95% confidence intervals (CI) are shown with lower limit and upper limit. *p*-value estimation is set with a significance < 0.05. Significant odds ratios are in bold.

League	Group Comparison	OR	Lower Limit 95% CI	Upper Limit 95% CI	*p*-Value
**Bundesliga**	Cat. 0 vs. Cat. 1	6.6500	1.7248	25.6390	**0.0059**
	Cat. 0 vs. Cat. 2	8.4444	2.1834	32.6587	**0.0020**
	Cat. 0 vs. Cat. 3	47.5000	4.6620	483.9645	**0.0011**
	Cat. 1 vs. Cat. 2	1.2698	0.4016	4.0156	0.6842
	Cat. 2 vs. Cat. 3	5.6250	0.6159	51.3758	0.1259
**Premier League**	Cat. 0 vs. Cat. 1	3.9286	0.3987	38.7051	0.2411
	Cat. 0 vs. Cat. 2	28.0000	2.8204	277.9723	**0.0044**
	Cat. 0 vs. Cat. 3	13.2632	1.5781	111.4692	**0.0173**
	Cat. 1 vs. Cat. 2	4.4000	0.9753	19.8513	0.0539
	Cat. 2 vs. Cat. 3	0.4737	0.1354	1.6570	0.2422
**Serie A**	Cat. 0 vs. Cat. 1	1.5476	0.4378	5.4706	0.4978
	Cat. 0 vs. Cat. 2	9.5333	2.4209	37.5418	**0.0013**
	Cat. 0 vs. Cat. 3	56.0769	2.8661	1097.1816	**0.0080**
	Cat. 1 vs. Cat. 2	6.1600	1.7377	21.8364	**0.0049**
	Cat. 2 vs. Cat. 3	6.6000	0.3377	128.9951	0.2134
**La Liga**	Cat. 0 vs. Cat. 1	1.1364	0.1077	11.9925	0.9153
	Cat. 0 vs. Cat. 2	5.7692	0.5949	55.9490	0.1305
	Cat. 0 vs. Cat. 3	10.8333	1.0281	114.1550	**0.0474**
	Cat. 1 vs. Cat. 2	5.0769	1.4951	17.2399	**0.0092**
	Cat. 2 vs. Cat. 3	1.8778	0.5546	6.3577	0.3112

**Table 3:** Detection and occurrence of soccer league COVID-19 cases for three different periods: Period 1—1 January 2020 until 31 December 2020; Period 2—1 January 2021 until last day of week 26, 2021); Period 3—first day of week 27, 2021 until end of study period. Ratio of cases to the overall detected cases per league occurring in the analyzed period. Mean case occurrence per week in the observed period studied.

League	Time/Period	Cases	Ratio of Cases	% of Cases	Mean Case/Week
**Bundesliga**	period 1	31	0.22	21.68	0.58
	period 2	18	0.13	12.59	0.69
	period 3	94	0.66	65.73	3.13
**Premier League**	period 1	40	0.44	44.44	0.75
	period 2	12	0.13	13.33	0.46
	period 3	38	0.42	42.22	1.27
**Serie A**	period 1	84	0.35	34.71	1.58
	period 2	46	0.19	19.01	1.77
	period 3	112	0.46	46.28	3.73
**La Liga**	period 1	45	0.36	35.71	0.85
	period 2	23	0.18	18.25	0.88
	period 3	58	0.46	46.03	1.93

## Data Availability

Not applicable.

## Supplementary Materials

The following supporting information can be downloaded at: https://www.mdpi.com/article/10.3390/life12081220/s1, Adnex S1; Odds ratio surrounding incidence with probability of occurrence of COVID-19 cases in the leagues.
