# Supplementary material for: COVID-19 in European Soccer: A Public 2-Year Comparison of COVID-19 Case Management and Case Characteristics between the 1st Bundesliga, La Liga, Serie A and the Premier League
Source: Life (Basel). 2022 Aug 11;12(8):1220. doi: 10.3390/life12081220 (PMC9409953; doi:10.3390/life12081220)
Supplement: Supplementary file 1 [file life-12-01220-s001.zip › life-1844820-supplementary.pdf]

Adnex S1: *Odds ratio surrounding incidence with probability of occurrence of COVID-19 cases in the leagues.*

Bundesliga				
Category	Incidence	Weeks with reported infection	Weeks without reported infection	Total
0	<50	4	19	23
1	50-199	14	10	24
2	200-499	16	9	25
3	>500	10	1	11
		44	39	

Premier League				
Category	Incidence	Weeks with reported infection	Weeks without reported infection	Total
0	<50	1	14	15
1	50-199	5	11	16
2	200-499	10	5	15
3	>500	18	19	37
		34	49	

Serie A				
Category	Incidence	Weeks with reported infection	Weeks without reported infection	Total
0	<50	6	13	19
1	50-199	10	14	24
2	200-499	22	5	27
3	>500	13	0	13
		51	32	

La Liga				
Category	Incidence	Weeks with reported infection	Weeks without reported infection	Total
0	<50	1	5	6
1	50-199	5	22	27
2	200-499	15	13	31
3	>500	13	6	19
		34	46	

Environmental national incidence for new Covid-19 cases was categorized for every week of the studied period related to ECDC data. For every week new reported cases in the related league were counted.
